# Supplementary material for: Quantitative Determination of 2-Oxo-Imidazole-Containing Dipeptides by High-Performance Liquid Chromatography/Tandem Mass Spectrometry
Source: Antioxidants (Basel). 2022 Dec 2;11(12):2401. doi: 10.3390/antiox11122401 (PMC9774565; doi:10.3390/antiox11122401)
Supplement: Supplementary file 1 [file antioxidants-11-02401-s001.zip › antioxidants-2025661-supplementary.pdf]

Supplementary Materials

Quantitative determination of 2-oxo-imidazole–containing dipeptides by high-performance liquid chromatography/tandem mass spectrometry

Somei Komae [1], Shingo Kasamatsu [1, 2], Koji Uchida [3], and Hideshi Ihara [1, 2, *]

[1] Department of Biological Science, Graduate School of Science, Osaka Prefecture University, 1-1 Gakuen-cho, Sakai 599-8531, Osaka, Japan

[2] Department of Biological Chemistry, Graduate School of Science, Osaka Metropolitan University, 1-1 Gakuen-cho, Sakai 599-8531, Osaka, Japan

[3] Graduate School of Agricultural and Life Sciences, The University of Tokyo, Tokyo 113-8657, Japan

* Corresponding author: Hideshi Ihara, Department of Biological Chemistry, Graduate School of Science, Osaka Metropolitan University, 1-1 Gakuen-cho, Sakai 599-8531, Osaka, Japan. Tel.: +81-72-254-9753; E-mail: iharah@omu.ac.jp

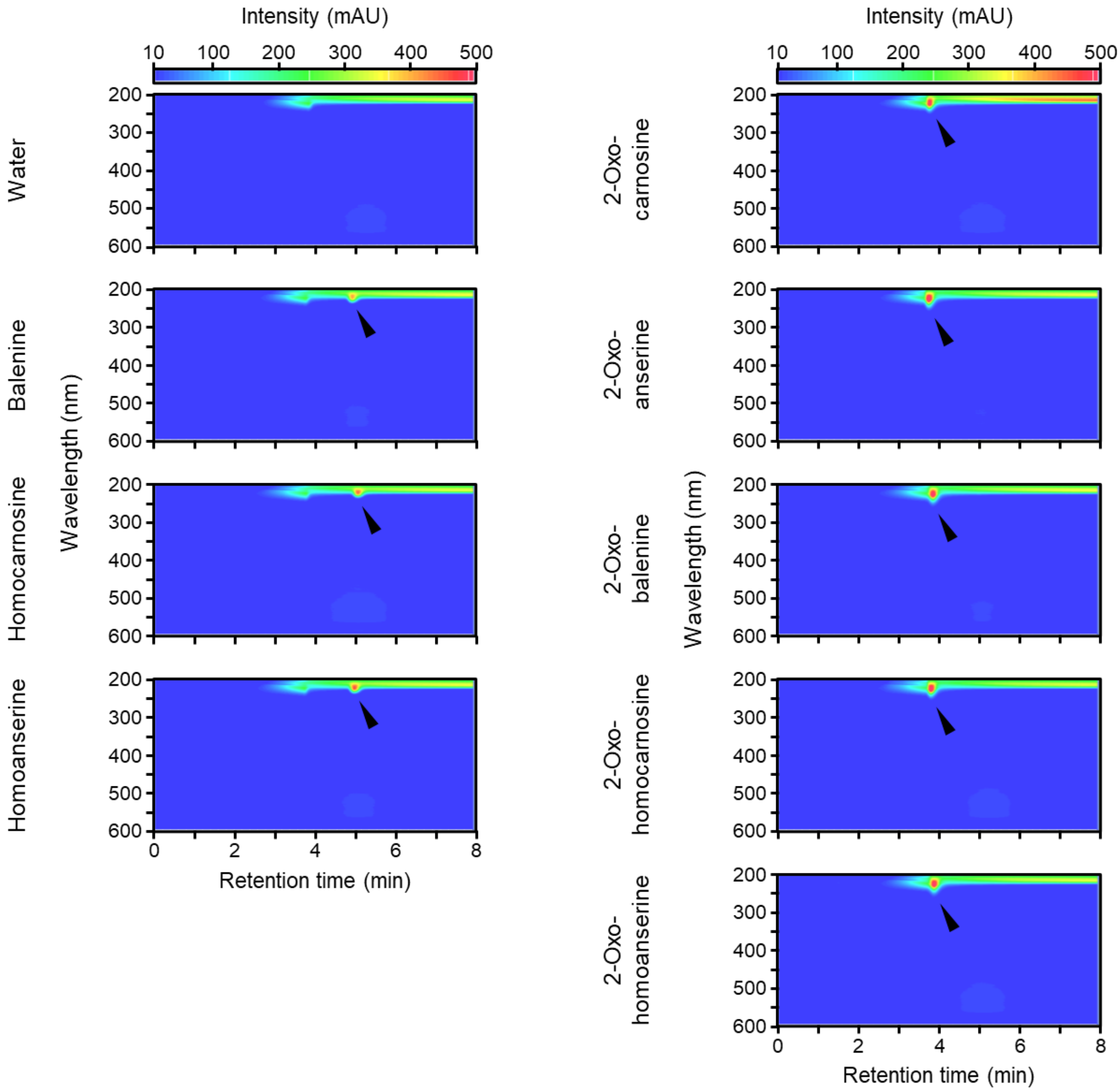

Supplementary Figure S1. PDA detection of synthesized IDPs and 2-oxo-IDPs. Synthesized IDPs and 2-oxo-IDPs (1 mM each) were separated by HPLC using a Scherzo SS-C18 column (2.0 mm × 50 mm; Imtakt) and the absorption spectra were recorded by a photodiode array (PDA) detector. Triangles indicate IDPs and 2-oxo-IDPs.

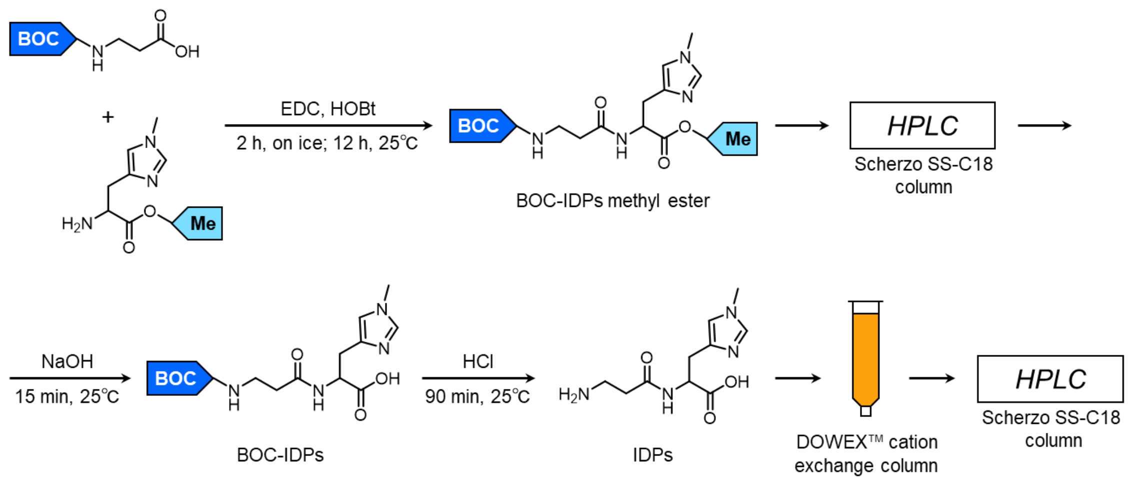

Supplementary Figure S2. Scheme of the preparation of IDPs. BOC-β-alanine was coupled with methyl ester derivatives of 1-methyl-histidine mediated by EDC and HOBt. Synthesized BOC-IDPs methyl ester derivatives were purified by HPLC using a Scherzo SS-C18 column. The eluate containing the synthesized compound was collected and reacted with NaOH and HCl to deprotect methyl ester and BOC groups, respectively. Crude IDPs were purified by DOWEX cation exchange column and HPLC using a Scherzo SS-C18 column. β-Alanine was replaced by GABA, and 1-methyl-histidine was replaced by histidine and 3-methyl-histidine in the synthesis of homocarnosine and homoanserine, respectively. The detailed information of this scheme is described in Materials and Methods section 2.2.2.

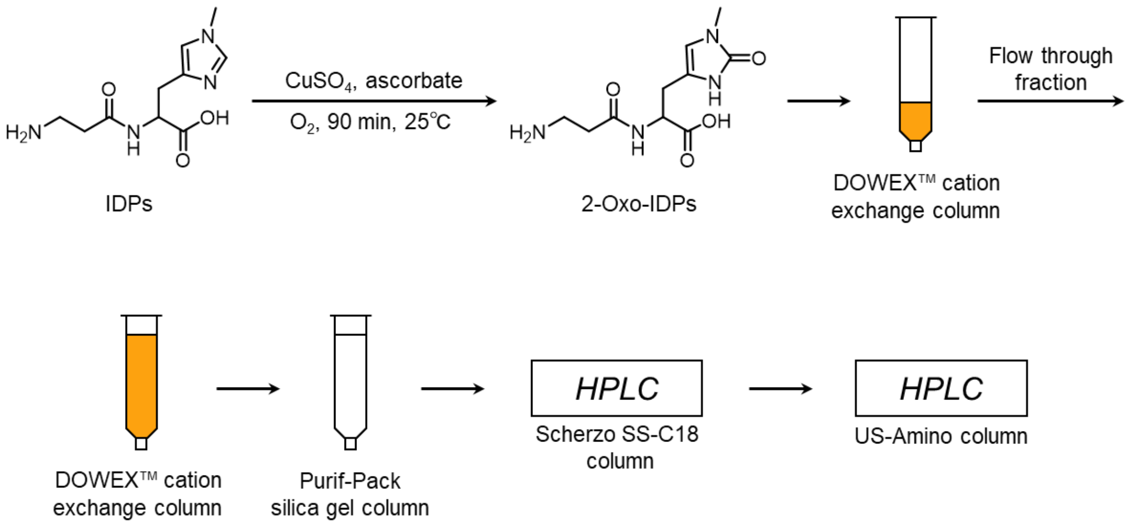

Supplementary Figure S3. Scheme of the preparation of 2-oxo-IDPs. IDPs were incubated with $CuSO_4$ and ascorbate under oxygen aeration, and the synthesized 2-oxo-IDPs were separated by 2 steps of DOWEX cation exchange column and silica gel column. Furthermore, 2-oxo-IDPs were purified by HPLC using a Scherzo SS-C18 and US-Amino column. The detailed information of this scheme is described in Materials and Methods section 2.3.

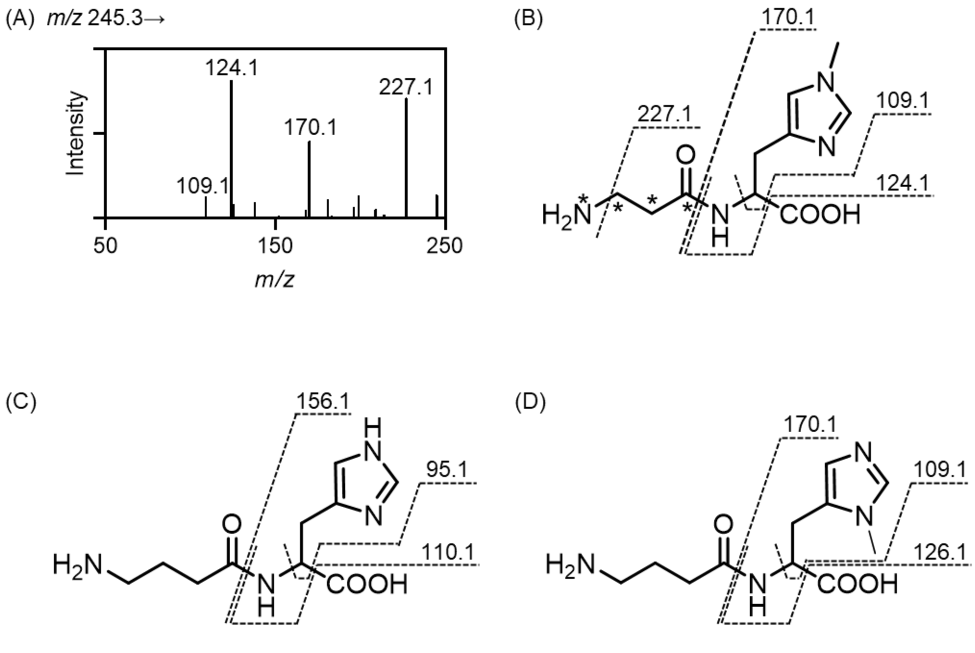

Supplementary Figure S4. HPLC-ESI-MS/MS analysis of stable isotope-labeled balenine and other IDPs. MS/MS spectra of stable isotope-labeled balenine (A), and the assignment of the product ions of stable isotope-labeled balenine (B), homocarnosine (C), and homoanserine (D). MS/MS spectra of homocarnosine and homoanserine are shown in Figure 1C and D. Asterisks indicate the position of ^{13}C and ^{15}N.

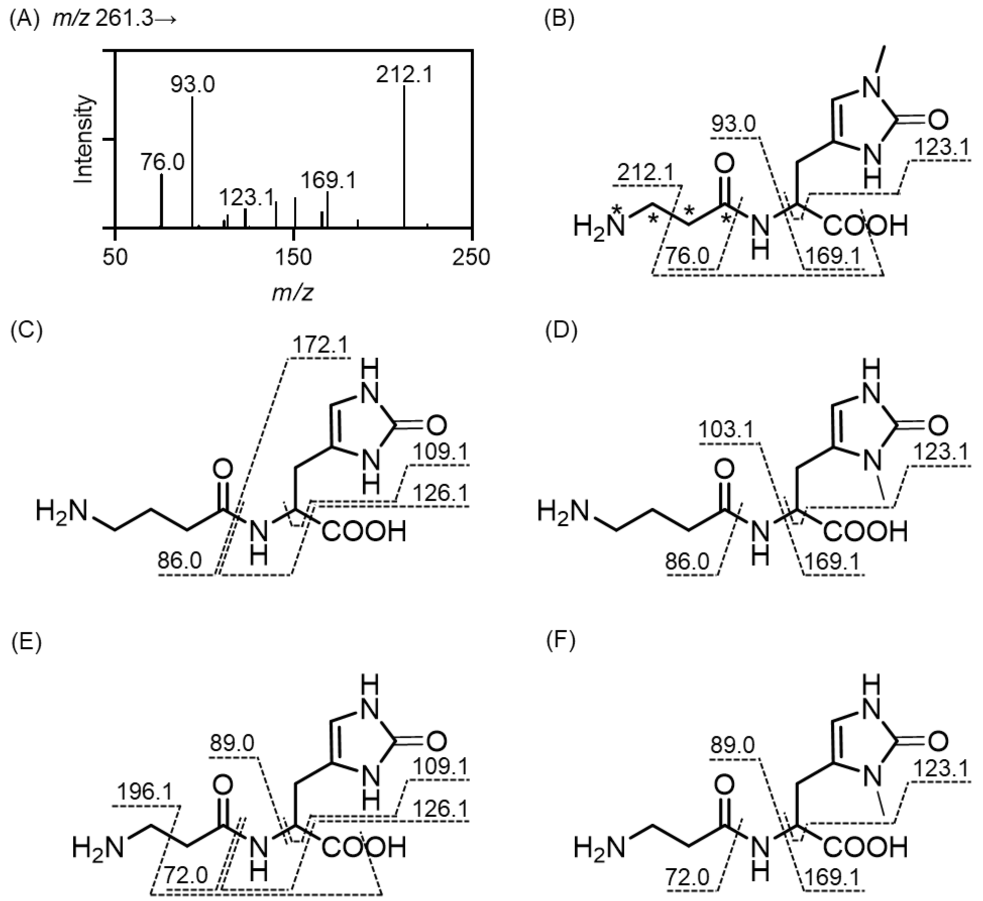

Supplementary Figure S5. HPLC-ESI-MS/MS analysis of isotope-labeled 2-oxo-balenine and other 2-oxo-IDPs. MS/MS spectra of stable isotope-labeled 2-oxo-balenine(A), and the assignment of the product ions of stable isotope-labeled 2-oxo-balenine (B), 2-oxo-homocarnosine (C), 2-oxo-homoanserine (D), 2-oxo-carnosine (E), and 2-oxo-anserine (F). MS/MS spectra of 2-oxo-homocarnosine, 2-oxo-homoanserine, 2-oxo-carnosine, and 2-oxo-anserine are shown in Figure 2C–F. Asterisks indicate the position of ^{13}C and ^{15}N.

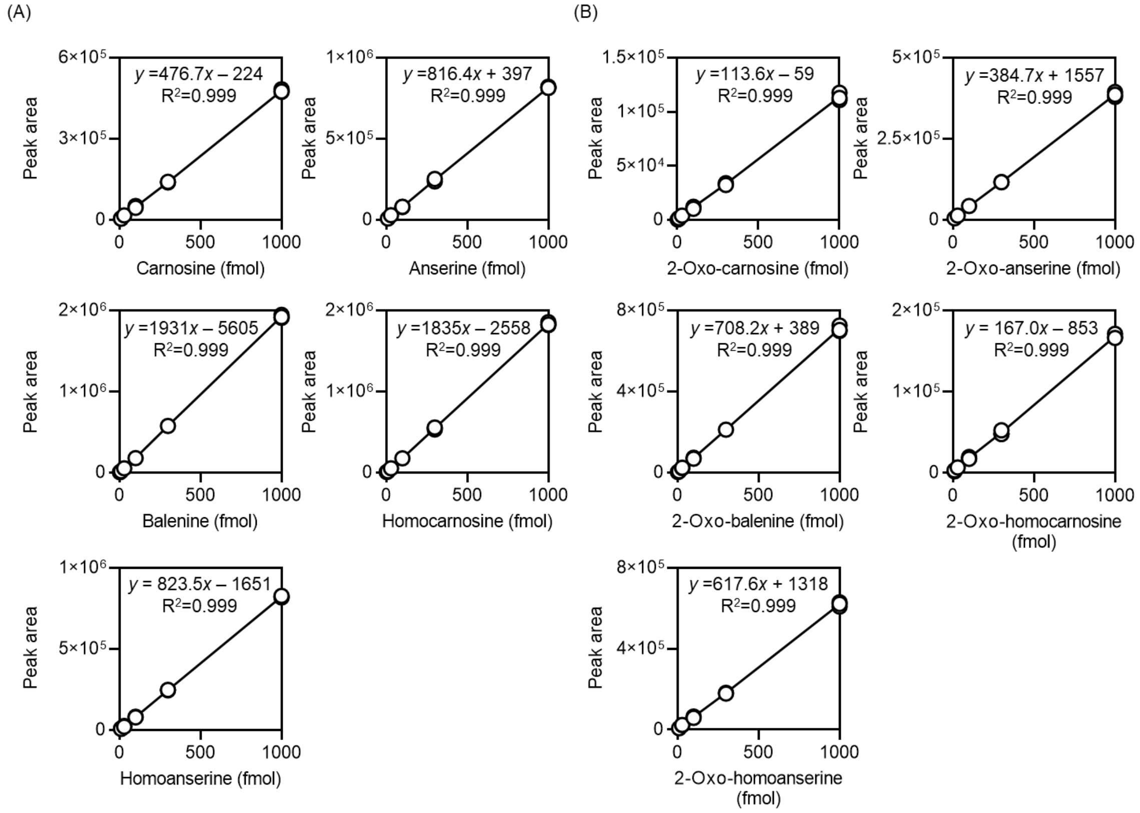

Supplementary Figure S6. Regression equation for the calibration curves of IDPs (A) and 2-oxo-IDPs (B). The peak areas of IDPs and 2-oxo-IDPs were obtained by HPLC-ESI-MS/MS and the standard calibration curve of IDPs and 2-oxo-IDPs were generated in the concentration range of 1–1000 fmol. Regression equations for the calibration curves were obtained using GraphPad Prism.
